# Effect of electroacupuncture on metabolic level and quality of life in patients with obese polycystic ovary syndrome: a randomized controlled trial

**DOI:** 10.3389/fendo.2025.1723419

**Published:** 2026-01-06

**Authors:** Yuqing Wang, Liying Fu, Jiqing Wang, Xiaoqin Fang, Shifen Xu, Shanshan Li, Yiqun Mi

**Affiliations:** Shanghai Municipal Hospital of Traditional Chinese Medicine, Shanghai University of Traditional Chinese Medicine, Acupuncture, Shanghai, China

**Keywords:** electroacupuncture, metabolic level, obese, polycystic ovary syndrome, quality of life, randomized controlled trial

## Abstract

**Objective:**

This study aims to investigate the effects of electroacupuncture (EA) intervention on patients with obese polycystic ovary syndrome (PCOS), with specific analysis focusing on the following dimensions: 1) whether EA can reduce patients’ body mass index (BMI); 2) to evaluate the regulatory effect of EA on key endocrine and metabolic indicators of patients, including blood glucose, blood lipids, and sex hormones; 3) to examine whether EA can improve reproduction-related outcomes in patients, such as abnormal menstrual cycles, abnormal menstrual flow, abnormal endometrial thickness, and ovulatory dysfunction; and 4) to verify the effect of EA in improving patients’ quality of life. Furthermore, this study intends to provide evidence-based support for the optimization of clinical non-pharmacological intervention strategies for obese PCOS.

**Methods:**

This was a patient- and assessor-blinded, randomized, sham-controlled trial, enrolling 106 female patients with obese PCOS aged 18–45 years. Participants were randomly assigned to two groups: the EA group and the sham EA group. The treatment duration was 12 weeks, with a 12-week follow-up period after treatment completion. The intervention frequency was adjusted in stages: three sessions/week during Weeks 1–4, two sessions/week during Weeks 5–8, and one session/week during Weeks 9–12, resulting in a total of 24 treatment sessions throughout the entire course. The primary outcome was the change in BMI from baseline to the end of the 12-week intervention. Secondary outcomes included changes in body composition, glucose metabolism indicators, lipid metabolism parameters, sex hormone levels, endometrial thickness, ovarian volume, menstrual cycle, and scores on the validated Chinese version of the Polycystic Ovary Syndrome Health-Related Quality of Life Questionnaire (PCOSQ).

**Results:**

Among the 106 patients (mean (SD) age, 27.93 (3.47) years) included in the intention-to-treat analysis, 104 (98%) completed all outcome measurements at week 24, and 2 (2%) dropped out of the trial. The mean difference in BMI from baseline to week 12 within the EA group was −2.46 (95%CI, −2.67 to −2.18). At week 12, the difference in BMI score was -1.50 (95%CI, -1.83 to -1.17;P<.001) between the EA and SA groups. The efficacy of EA was sustained during the 24-week post-intervention follow-up (mean difference, -1.58;95% CI: -2.05 to -1.10; P<.001). Significant improvements were also observed in the waist-hip ratio (WHR) and body fat mass (BFM). The mean difference in WHR and BFM from baseline to week 12 within the EA group were -0.04 (95%CI, −0.06 to −0.04) and -4.56 (95%CI, −5.10 to −4.02). At week 12, the difference in WHR score was -0.03 (95%CI, -0.04 to -0.01;P<.001) between the EA and SA groups, in BFM was -2.92 (95%CI, -3.52 to -2.33;P<.001). For sex hormones, the mean difference in prolactin (PRL) and estradiol (E2) from baseline to week 12 within the EA group were -109.10 (95%CI, -154.60, -62.63) and 12.99 (95%CI, -17.45, 43.43), respectively. Regarding glucose metabolism, the mean difference in fasting glucose (FPG), fasting insulin (FINS), and calculated insulin resistance (HOMA-IR) from baseline to week 12 within the EA group were -0.30 (95%CI, -0.41, -0.18), -46.03(95%CI, -61.27, -30.79), and -12.70 (95%CI, -18.58, -6.81), respectively. For lipid profiles, the mean difference in total cholesterol (TC), triglycerides (TG), low-density lipoprotein cholesterol (LDL-C) from baseline to week 12 within the EA group were -0.30 (95%CI, -0.40, -0.20), -0.44 (95%CI, -0.64,-0.25), and -0.30 (95%CI, -0.43, -0.16), respectively. PCOSQ improvements from baseline consistently favored EA at all timepoints. No serious adverse events were reported. No serious adverse events were reported.

**Conclusion:**

Electroacupuncture is an effective intervention for managing weight, improving metabolism and enhancing the quality of life in patients with obese PCOS. The benefits of BMI and PCOSQ last for at least 24 weeks.

**Clinical Trial Registration:**

https://www.chictr.org.cn, identifier ChiCTR2300070722.

## 1 Introduction

Polycystic ovary syndrome (PCOS) is a prevalent endocrine disorder. Its initial symptoms include reduced menstrual flow, which progressively manifests as acne and obesity, and may ultimately lead to infertility. Severe cases may lead to chronic mood disturbances (1, 2). Epidemiological studies indicate that PCOS affects 6% to 25% of reproductive-aged women globally (3). with a significantly higher prevalence of 41.3% among obese individuals (4). Obesity exacerbates glycolipid metabolic dysregulation in PCOS (5), and intensifies ovarian dysfunction, thereby elevating the risks of metabolic syndrome and atherosclerosis (6). Consequently, weight management is critical for obese PCOS.

Current clinical guidelines universally recommend lifestyle modification as first-line treatment for obese PCOS patients (7). However, poor patient compliance necessitates adjunct pharmacological interventions, such as oral contraceptives or ovulation induction agents. These treatments are frequently associated with adverse effects (e.g. nausea, vomiting, bloating, headaches, and weight gain) (8, 9), highlighting the need for safer and more effective alternative treatments for obese PCOS.

Acupuncture, recognized for its efficacy, cost-effectiveness and minimal invasiveness, has demonstrated promising outcomes in PCOS management (10, 11). Based on the results of the network meta-analysis, acupuncture was associated with the lowest net cost compared to other therapies in the health economic evaluation of PCOS treatments. It represents the most cost-effective option that delivers both clinical efficacy and economic benefit (12). Jiaman Wu’s study shows that acupuncture can regulate serum hormone levels and restore ovarian ovulation function in PCOS infertility patients. Caina Liu’s research also confirms this (10, 13). Additionally, Qi Huang’s study shows that acupuncture can improve obesity and significantly increase insulin sensitivity in PCOS patients (14). Despite this, high-quality randomized controlled trials focusing on electroacupuncture (EA) as a primary intervention remain scarce. As a therapeutic method combining traditional acupuncture with modern electrophysiological technology, EA provides sustained, quantifiable electrical stimulation, which has demonstrated more consistent and potent neuromodulatory effects on the hypothalamic-pituitary-ovarian axis and metabolic pathways compared to manual needling alone. Previous studies have indicated that EA at specific acupoints can improve insulin resistance in rat models of PCOS by promoting the browning of white adipose tissue. This study therefore aimed to evaluate the therapeutic efficacy and incidence of adverse reactions of EA in patients with obese PCOS using a randomized, placebo-controlled design, following rigorous methodological standards. The objective was to provide a safe and effective alternative treatment option for this patient population.

## 2 Methods

### 2.1 Study design

This randomized sham-controlled clinical trial was conducted from July 2023 to October 2024, at the Shanghai Municipal Hospital of Traditional Chinese Medicine, China. This trial was registered (ChiCTR2300070722) and the protocol was approved by the Ethics Committee of Shanghai Hospital of Traditional Chinese Medicine (2023SHL-KY-79-01). Written informed consent was obtained from participants before enrollment. This study followed the Consolidated Standards of Reporting Trials (CONSORT) reporting guideline (15).

### 2.2 Participants

Participants were eligible if they were women aged 18–45 years, who met the diagnostic criteria for PCOS diagnosis, had a body mass index (BMI) ≥25kg/m^2^, and demonstrated evidence of insulin resistance (HOMA-IR≥2.69) or laboratory-confirmed dysglycemia, dyslipidemia, sex hormone abnormalities or sex hormone-related drug therapy with in the last 2 months, and were willing to provide written informed consent. The exclusion criteria included hyperandrogenism or ovulatory dysfunction attributable to non-PCOS etiologies, such as premature ovarian insufficiency, thyroid disorders or hyperprolactinemia; diagnosis of severe cardiac, hepatic, renal, or psychiatric comorbidities; and patients refusal to participate.

### 2.3 Randomization and masking

Eligible participants were randomly assigned in a 1:1 ratio to the EA or SA group using a computer-generated simple randomization sequence. The sequence was created and the sequence was generated by an independent researcher who was blinded to the group assignment. Allocation concealment was ensured through sequentially numbered, opaque, sealed envelopes. Due to the nature of acupuncture interventions, practitioners performing acupuncture procedures were necessarily unblinded to group assignments. However, rigorous blinding was implemented for participants, outcome assessors and data statisticians.

### 2.4 Intervention

All participants received standardized lifestyle health education upon enrollment, which includes diet, exercise, and daily behavior management. **Supplementary Data Sheet 1** describes the recommendations for diet, exercise, and daily behavioral habits. Timely supervision and guidance for lifestyle interventions were provided to patients on the WeChat platform.

The semi-standardized treatment scheme was applied based on the clinical experience. Both EA and SA were performed by licensed acupuncturists with at least 2 years of experience. Standardized operating procedures were established for acupuncturists and the training content included the precise location of acupoints, choice of needle type based on acupoints, needle manipulation techniques, and standardized communication protocols with patients. 24 sessions of EA or SA were delivered over 12 weeks, with treatment frequency at 3 times for 4 weeks, twice a week for 4 weeks and once a week for 4 weeks. Location of acupoints followed the World Health Organization’s Standard Acupuncture Locations (16). The main acupoints were Guanyuan (RN4), Uterus (EX-CA1), Guilai (ST29), Sanyinjiao (SP6) and Taixi (KI3) and additional acupoints were chosen by the acupuncturists based on patients’ syndrome differentiation during each session of treatment. When patients’ tongue exhibits dark purple with possible bruising, and the pulse is deep and rough, choose acupoints for the Kidney Deficiency and Blood Stasis Type. When patients’ tongue exhibits slightly red or dim, with a thin white or slightly yellow coating, and the pulse is wiry and thin, choose acupoints for Kidney Deficiency and Liver Depression Type. When patients’ tongue exhibits swollen, with tooth marks on the edges, and the pulse is soggy and slow, choose acupoints for the Spleen Deficiency Phlegm Dampness Type. (**Supplementary Figure S1**; **Supplementary Table S1**). Every treatment session lasted for 30 min in a private quiet space, with each participant wearing an eye-patch and in a lying position. All manipulation should adhere to the Standards for Reporting Interventions in Clinical Trials of Acupuncture (STRICTA) guideline (17).

#### 2.4.1 Electroacupuncture group

For the EA group, acupuncturists used disposable stainless-steel needles of varying sizes depending on the acupoint (Wuxi Jiajian Medical Material Co., Ltd. Wuxi, China, 0.25*40mm). Following the insertion of the needle to a certain depth at specific acupoints, manual manipulation techniques such as lifting-thrusting or rotating were applied to elicit the Deqi sensation. Once patients received Deqi, then the EA device (CMNS6-1, Wuxi Jiajian Medical Device Co., Ltd., China) was applied, two pairs of electrodes connecting to bilateral EX-CA1 and ST29, delivering a continuous wave based on the patient’s tolerance. **Supplementary Data Sheet 1** details the basis for setting EA parameters.

#### 2.4.2 Sham Electroacupuncture group

For the SA group, acupuncturists used a tipped placebo needle named Streitberger placebo needle from Germany (18). No manipulation was performed with no attempt to induce the Deqi sensation. The same model of EA device was connected to the identical acupoints as in the EA group. All parameters of the EA device were set to 0, with only the light turned on. The duration and frequency of this sham treatment were kept consistent with those of the EA group. **Supplementary Data Sheet 1** explains the rationale for establishing the SA group.

### 2.5 Outcomes

The primary outcome was BMI measured at week 12. The height and weight of the patients were measured at baseline, at 12 weeks, and 24 weeks to calculate BMI. A higher BMI indicates a higher degree of obesity and a greater health risk.

The secondary outcomes included waist-hip ratio (WHR), body fat mass (BFM), skeletal muscle mass (SMM), Basal Metabolic Rate (BMR), glucose metabolism indicators as fasting glucose (FPG), two-hour postprandial blood glucose (2hPG), fasting insulin (FINS), and calculated insulin resistance (HOMA-IR); lipid metabolism indicators as total cholesterol (TC), triglycerides (TG), low-density lipoprotein cholesterol (LDL-C), and high-density lipoprotein cholesterol (HDL-C); sex hormone indicators including as follicle-stimulating hormone (FSH), luteinizing hormone (LH), prolactin (PRL), T, and estradiol (E2); endometrial thickness and ovarian volume (OV), and the days of menstrual length. These indicators were measured at baseline and at week 12. The participants completed the Polycystic Ovary Syndrome Health-Related Quality of Life Questionnaire (PCOSQ) at week 4, week 8, week 12, week 16, week 20, and week 24. This questionnaire comprises 5 dimensions: mood, body hair, weight, infertility, and menstrual problems. The PCOSQ total score ranges from 26 to 182, where a higher score indicates a better quality of life for PCOS patients.

Adverse events were evaluated following each treatment session through direct inquiry with participants, and identified via patient self-reports during the trial process as well. All adverse events were categorized based on their relevance to the treatment and were addressed in a timely manner.

### 2.6 Assessment of blinding success

After the completion of treatment, an independent evaluator will conducted an assessment of the success of blinding implementation via telephone interview. The following core question was recorded in the Case Report Form: “When you voluntarily participated in this study, you were informed that you had an equal chance of receiving EA or SA treatment. Now that the study has been completed, which type of treatment do you think you received?” Three options will be provided to the participants: EA treatment, SA treatment, and unsure. Statistical analysis will be performed on the participants’ choices. Using the Bang blinding index, a directional score within the range of (–1, 1) can be calculated: a positive score indicates that the participant correctly guessed the group allocation, i.e., blinding implementation failed; a negative score indicates that the participant incorrectly guessed the group allocation, i.e., blinding implementation succeeded.

### 2.7 Sample size calculation and statistical analysis

In this trial, the sample size calculation was based on a clinical study (19). We assumed that EA group would outperform the SA group by 2.25 points kg/m² in the BMI score, with a standard deviation (SD) of 3.7 for the both groups. A sample size of 84 was required to achieve 90% power with a 2-sided α of 0.05. Considering a dropout rate of 20%, a total of 106 participants (53 participants for each group) were needed.

An intention-to-treat (ITT) approach was used and all randomly assigned participants were analyzed. Continuous variables were described as mean (SD) or median (p25, p75). In this study, linear mixed-effects models (LMMs) were employed to analyze the repeated-measures data of BMI and PCOSQ scores. The model specifications are detailed as follows: Fixed effects included treatment group, time point, the interaction term between treatment group and time point, and baseline measurements as covariates. The treatment groups were categorized into the EA group and the SA group. Time points were treated as within-subject repeated-measurement factors, and the interaction term was used to evaluate intergroup differences in temporal changes. Random effects were specified as subject-specific random intercepts based on participant IDs to account for interindividual variability and control for the correlation among repeated measurements. Given the limited number of measurement time points and the need to ensure model convergence, random slopes for time points were not included. Model comparisons based on the Akaike Information Criterion (AIC) supported this more parsimonious random-effect structure. Regarding the residual covariance structure, an unstructured (UN) matrix was adopted for BMI data with only three time points, while a first-order autoregressive (AR(1)) structure was selected for PCOSQ data with seven time points based on superior model fit indices. Parameter estimation was performed using restricted maximum likelihood (REML). Hypothesis testing for fixed effects utilized the Kenward-Roger (KR) degrees of freedom approximation to enhance the robustness of inferences in small samples. The significance level was set at 0.05, and *post-hoc* comparisons were conducted with Tukey’s method for multiple comparison correction. The differences between group for WHR, BFM, SMM, VFL, glucose metabolism and indicators, lipid metabolism indicators, sex hormone index, endometrial thickness and OV, and the days of menstrual cycle were assessed using the analysis of covariance or the Mann-Whitney U test. Within-group pre-post treatment differences were analyzed with the Wilcoxon signed-rank test or the paired t-tests. All statistical analyses were performed by an independent statistician using SPSS 26.0. Two-sided P values less than 0.05 were considered statistically significant. To control the inflation of Type I error caused by multiple comparisons, this study adopted a hierarchical correction strategy based on analytical priority: Secondary outcomes were first pre-classified into 5 analysis domains according to clinical physiological mechanisms, with independent Bonferroni correction applied within each domain. Specifically, the significance thresholds were set as follows: 0.0125 (0.05/4) for body composition (4 indicators), glucose metabolism (4 indicators), and lipid metabolism (4 indicators); 0.01 (0.05/5) for sex hormones (5 indicators); and 0.017 (0.05/3) for ovarian function (3 indicators).

## 3 Results

### 3.1 Participant characteristics

**Figure 1:**
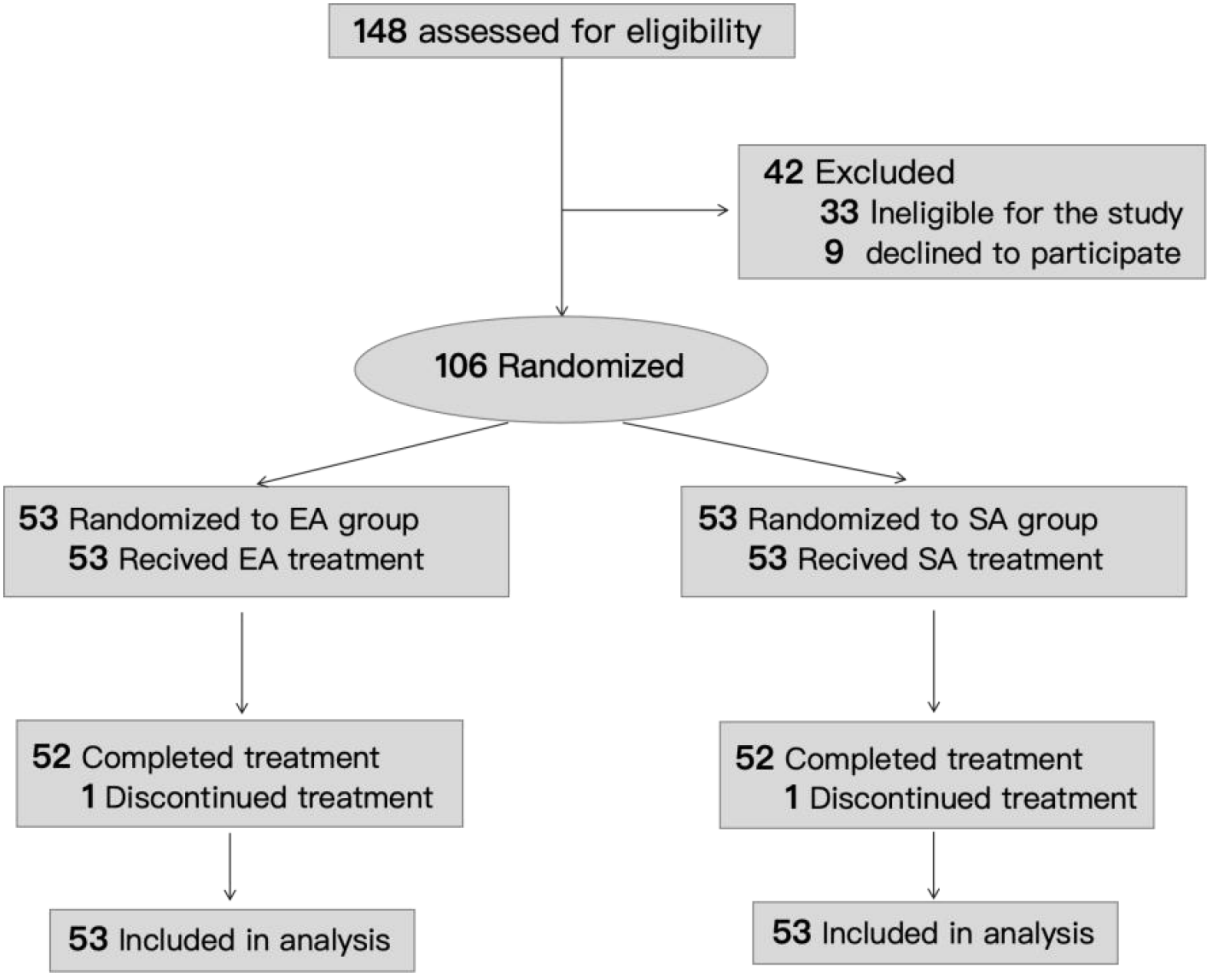
Flow of participants through the trial.

From July 2023 to October 2024, 148 participants were screened, and 106 were enrolled at baseline. 42 participants were excluded before randomization: 33 were ineligible for the study and 9 refused to sign the informed consent form. During the treatment, two participants were dropped out due to job changes(one in the EA group, one in the SA group). This study used ITT analysis, so a total of 106 patients were included in the statistical analysis (**Figure 1**).

The sociodemographic and clinical characteristics, and baseline outcomes were similar between the 2 groups (**Table 1**). The mean age was 27.9 (3.81) years in the EA group, 28(3.15) years in the SA group; the mean course of the disease was 67.90(58.50) month in the EA group, 58.30(48.20) month in the SA group; the mean height was 162(5.26) cm in the EA group, 163(5.63) cm in the SA group; the mean weight was 74.90(9.38) kg in the EA group, 71.70(8.76) kg in the SA group. The mean BMI was 28.19 (2.98) kg/m² in the EA group and 27.71 (2.69) kg/m² in the SA group; the mean WHR was 0.93 (0.06) in the EA group and 0.93 (0.05) in the SA group; the mean BFM was 30.70 (5.39) kg in the EA group and 29.70 (5.13) kg in the SA group; the mean SMM was 25.40 (2.95) kg in the EA group and 24.60 (3.08) kg in the SA group; the mean BMR was 1350 (116) kJ/(h·m²) in the EA group and 1329 (121) kJ/(h·m²) in the SA group; the median FPG [M(P25, P75)] was 5.40 (5.00, 5.70) mmol/L in the EA group and 5.30 (5.00, 5.65) mmol/L in the SA group; the median 2hPG [M(P25, P75)] was 6.20 (5.30, 6.95) mmol/L in the EA group and 6.30 (5.55, 6.70) mmol/L in the SA group; the median FINS [M(P25, P75)] was 95.10 (55.25, 175.50) pmol/L in the EA group and 86.80 (56.10, 170.00) pmol/L in the SA group; the median HOMA-IR [M(P25, P75)] was 23.66 (12.64, 45.37) in the EA group and 19.44 (14.86, 30.07) in the SA group; the mean TC was 5.08 ± 0.91 mmol/L in the EA group and 5.09 ± 0.85 mmol/L in the SA group; the median TG [M(P25, P75)] was 1.55 (0.80, 2.06) mmol/L in the EA group and 1.14 (0.88, 1.60) mmol/L in the SA group; the mean LDL-C was 3.18 ± 0.74 mmol/L in the EA group and 3.19 ± 0.54 mmol/L in the SA group; the median HDL-C [M(P25, P75)] was 1.34 (1.23, 1.53) mmol/L in the EA group and 1.41 (1.21, 1.65) mmol/L in the SA group; the median FSH [M(P25, P75)] was 5.49 (4.62, 6.64) mIU/L in the EA group and 5.46 (4.67, 6.86) mIU/L in the SA group; the median LH [M(P25, P75)] was 6.83 (4.82, 9.46) mIU/L in the EA group and 7.88 (4.10, 9.66) mIU/L in the SA group; the median PRL [M(P25, P75)] was 326.00 (208.59, 415.16) mIU/L in the EA group and 300.40 (207.85, 406.07) mIU/L in the SA group; the median T [M(P25, P75)] was 1.53 (1.27, 1.90) nmol/L in the EA group and 1.54 (1.32, 1.92) nmol/L in the SA group; the median E2 [M(P25, P75)] was 138.00 (104.00, 280.50) pmol/L in the EA group and 141.00 (108.50, 202.00) pmol/L in the SA group; the median Endometrial Thickness [M(P25, P75)] was 6.00 (5.44, 7.30) mm in the EA group and 6.00 (5.10, 6.79) mm in the SA group; the median OV [M(P25, P75)] was 13.45 (12.43, 14.04) ml in the EA group and 13.10 (12.55, 14.01) ml in the SA group; the mean Days of Menstrual Cycle was 44.64 ± 22.15 days in the EA group and 46.85 ± 19.56 days in the SA group; the mean PCOSQ score was 96.38 (25.23) in the EA group and 95.91 (21.64) in the SA group.

**Table 1:** Baseline characteristics of study participants.

Characteristics	EA (n=53)	SA (n=53)
Mean age (SD), y	27.90 (3.81)	28 (3.15)
Mean Duration of disease (SD), m	67.90 (58.50)	58.30 (48.20)
Height (SD), cm	162 (5.26)	163 (5.63)
Weight (SD), kg	74.90 (9.38)	71.70 (8.76)
BMI (SD), kg/m^2^	28.19 (2.98)	27.71 (2.69)
WHR (SD)	0.93 (0.06)	0.93 (0.05)
BFM (SD), kg	30.70 (5.39)	29.70 (5.13)
SMM (SD), kg	25.40 (2.95)	24.60 (3.08)
BMR (SD), kJ/h·m^2^	1350 (116)	1329 (121)
FPG[M (P25, P75)], mmol/L	5.40 (5.00, 5.70)	5.30 (5.00, 5.65)
2hPG[M (P25, P75)], mmol/L	6.20 (5.30, 6.95)	6.30 (5.55, 6.70)
FINS[M (P25, P75)], pmol/L	95.10 (55.25, 175.50)	86.80 (56.10, 170.00)
HOMA-IR[M (P25, P75)]	23.66 (12.64, 45.37)	19.44 (14.86, 30.07)
TC (SD), mmol/L	5.08 ± 0.91	5.09 ± 0.85
TG[M (P25, P75)], mmol/L	1.55 (0.80, 2.06)	1.14 (0.88, 1.60)
LDL-C (SD), mmol/L	3.18 ± 0.74	3.19 ± 0.54
HDL-C[M (P25, P75)], mmol/L	1.34 (1.23, 1.53)	1.41 (1.21, 1.65)
FSH[M (P25, P75)], mIU/L	5.49 (4.62, 6.64)	5.46 (4.67, 6.86)
LH[M (P25, P75)], mIU/L	6.83 (4.82, 9.46)	7.88 (4.10, 9.66)
PRL[M (P25, P75)], mIU/L	326.00 (208.59, 415.16)	300.40 (207.85, 406.07)
T[M (P25, P75)], nmol/L	1.53 (1.27, 1.90)	1.54 (1.32, 1.92)
E2[M (P25, P75)], pmol/L	138.00 (104.00, 280.50)	141.00 (108.50, 202.00)
Endometrial Thickness[M (P25, P75)], mm	6.00 (5.44, 7.30)	6.00 (5.10, 6.79)
OV[M (P25, P75)], ml	13.45 (12.43, 14.04)	13.10 (12.55, 14.01)
Days of Menstrual Cycle (SD), day	44.64 ± 22.15	46.85 ± 19.56
PCOSQ (SD), score	96.38 (25.23)	95.91 (21.64)

EA, Electroacupuncture; SA, Sham Electroacupuncture; BMI, Body Mass Index; WHR, Waist-Hip Ratio; BFM, Body Fat Mass; SMM, Skeletal Muscle Mass; BMR, Basal Metabolic Rate; FPG, Fasting Plasma Glucose; 2hPG, 2-Hour Postprandial Plasma Glucose; FINS, Fasting Insulin; HOMA-IR, Homeostasis Model Assessment of Insulin Resistance; TC, Total Cholesterol; TG, Triglycerides; LDL-C, Low-Density Lipoprotein Cholesterol; HDL-C, High-Density Lipoprotein Cholesterol; FSH, Follicle-Stimulating Hormone; LH, Luteinizing Hormone; PRL, Prolactin; T, Testosterone; E2, Estradiol; OV, Ovarian Volume; PCOSQ, Polycystic Ovary Syndrome Health-Related Quality of Life Questionnaire.

### 3.2 Primary outcome

Following 12-week treatment, the mean(SD) BMI was 25.76 (2.68) in the EA group, 26.76 (2.41) in the SA group. Following 24-week follow-up, the mean BMI was 24.70 (2.07) in the EA group, 25.97 (2.15) in the SA group. EA produced significantly reductions in BMI compared to SA, with the between-group differences of -1.51 (95% CI, -1.83 to -1.18; P<.001) at 12-week and -1.58 (95% CI, -2.05 to -1.10; P<.001) at 24-week (**Table 2**; **Supplementary Figure S2**).

**Table 2:** Primary and secondary outcomes.

Outcome	EA (n=53)		SA (n=53)		Difference between groups *Z/t*	*P*
	Mean change from baseline (95%CI)	*P*	Mean change from baseline (95%CI)	*P*		
Primary Outcome						
BMI						
Intervention phase						
Week 12	-2.46 (-2.67, -2.18)		-0.95 (-1.14, -0.75)			<.001
Follow-up phase						
Week 12	-3.49 (-3.85, -3.13)		-1.73 (-2.01, -1.46)			<0.001
Secondary Outcomes						
WHR						
Week 12	-0.04 (-0.06, -0.04)		-0.02 (-0.02, -0.01)			<0.001*
BFM						
Week 12	-4.56 (-5.10, -4.02)		-1.64 (-1.94, -1.33)			<0.001*
SMM						
Week 12	0.52 (-0.06, 1.10)		-0.01 (-0.60, 0.5)			0.205
BMR						
Week 12	29.26 (4.63, 53.9)		-6.68 (-34.78, 21.42			0.056
Glucose Metabolism Indicators						
FPG (mmol/L)						
Week 12	-0.30 (-0.41, -0.18)	<0.001	-0.10 (-0.19, -0.00)	0.028	-1.80	0.008*
2hPG (mmol/L)						
Week 12	-0.04 (-0.22, 0.14)	0.372	-0.10 (-0.26, 0.19)	0.758	-0.91	0.977
FINS (pmol/L)						
Week 12	-46.03 (-61.27, -30.79)	<0.001	-4.59 (-9.20, 0.02)	0.019	-2.10	<0.001*
HOMA-IR						
Week 12	-12.70 (-18.58, -6.81)	<0.001	-1.55 (-3.40, -0.06)	0.014	-1.89	<0.001*
Lipid Metabolism Indicators						
TC (mmol/L)						
Week 12	-0.30 (-0.40, -0.20)	<0.001	-0.07 (-0.27, 0.13)	0.491	-2.10	0.034
TG (mmol/L)						
Week 12	-0.44 (-0.64,-0.25)	<0.001	-0.01 (-0.10, 0.07)	0.735	-0.89	<0.001*
LDL-C (mmol/L)						
Week 12	-0.30 (-0.43, -0.16)	<0.001	-0.07 (-0.18, 0.04)	0.235	-2.04	0.011*
HDL-C (mmol/L)						
Week 12	-0.00 (-0.04, 0.04)	0.802	-0.02 (-0.09, -0.04)	0.846	-1.66	0.529
Sex Hormone Index						
FSH (mIU/L)						
Week 12	0.48 (0.09, 0.86)	0.038	0.08 (-0.33, 0.49)	0.015	-1.01	0.160
LH (mIU/L)						
Week 12	-0.64 (-1.61, 0.32)	0.185	-2.53 (-6.92, 1.85)	0.609	-0.66	0.393
PRL (mIU/L)						
Week 12	-109.10 (-154.60, -62.63)	<0.001	–40.27 (-79.86, -0.68)	0.066	-1.99	0.024
T (nmol/L)						
Week 12	-0.17 (-0.26, -0.07)	<0.001	-0.06 (-0.16, 0.04)	0.098	-0.41	0.117
E2 (nmol/L)						
Week 12	12.99 (-17.45, 43.43)	0.256	-46.14 (-95.60, 3.32)	0.170	-2.69	0.042
Endometrial Thickness and Ovarian Volume						
Endometrial Thickness (mm)						
Week 12	1.83 (1.31, 2.35)	<0.001	2.10 (1.57, 2.62)	<0.001	-0.06	0.471
OV (ml)						
Week 12	-4.05 (-4.49, -3.62)	<0.001	-3.89 (-4.22, -3.55)	<0.001	-0.50	0.544
Days of Menstrual Cycle (day)						
Week 12	-6.50 (-9.13, -3.87)	<0.001	-7.39 (-10.86, -3.91)	<0.001	-0.93	0.353
PCOSQ (score)						
Intervention phase						
Week 4	15.43 (11.82, 19.05)		9.30 (6.24, 12.37)		2.59	0.011
Week 8	20.66 (16.05, 25.28)		11.98 (8.54, 15.42)		3.03	0.03
Week 12	26.70 (22.03, 31.37)		16.19 (12.15, 20.23)		3.42	<0.001
Follow-up phase						
Week 16	29.81 (25.20, 34.43)		19.34 (15.26, 23.72)		3.41	<0.001
Week 20	33.58 (28.68, 38.49)		21.09 (13.47, 25.72)		3.72	<0.001
Week 24	36.58 (31.41, 41.76)		22.11 (17.41, 26.81)		4.15	<0.001

* indicates that after Bonferroni correction, the P-values for intergroup comparisons remain statistically significant (P < 0.05/number of comparisons; see the Methods section for details on the exact number of comparisons).

### 3.3 Secondary outcomes

The WHR and BFM were significantly decreased in the EA group compared with the SA with differences of -0.03 (95% CI, -0.04 to -0.01;P<.001) and -2.92 (95% CI, -3.52 to -2.33; P<.001), respectively. No between-group differences were observed in SMM and BMR (**Table 2**; **Supplementary Figure S2**).

The glucose metabolism parameters were significantly lower after 12-week treatment in the EA group than in the SA group except 2hPG (P<0.01), with the mean change from baseline in FPG, FINS, and HOMA-IR indicators of -0.30 (95% CI, -0.41 to -0.18; P<.001), -46.03 (95% CI, -61.27 to -30.79; P<.001), and -12.70 (95% CI, -18.58 to -6.81; P<.001),respectively (**Table 2**; **Supplementary Figure S2**).

The lipid profile markers were significantly lower after 12-week treatment in the EA group than in the SA group except HDL-C (P<0.05), with the mean change from baseline in TC, TG, and LDL-C indicators of -0.30 (95% CI, -0.40 to -0.20; P<.001), -0.44 (95% CI, -0.64 to -0.25; P<.001), and -0.30 (95% CI, -0.438 to -0.161; P<.001),respectively (**Table 2**; **Supplementary Figure S2**).

Regarding sex hormone modulation, after 12 weeks of treatment, the PRL level in the EA group was significantly decreased, while the E2 level was slightly increased. The mean changes from baseline were -109.10 (95% CI, -154.60 to -62.63, P < 0.001) and 12.99 (95% CI,-17.45 to 43.43, P = 0.256), respectively (**Table 2**; **Supplementary Figure S2**). Despite the mean E2 level in the EA group increasing by 12.99 nmol/L compared with the baseline, its 95% CI crossed zero (-17.45 to 43.43 nmol/L), indicating no statistically significant difference in E2 levels within the EA group before and after treatment.

Furthermore, Endometrial thickness, OV, and days of menstrual cycle showed significant within-group improvements in both groups by 12 weeks (P<.001), but no significant between-group differences were observed (**Table 2**; **Supplementary Figure S2**).

Notably, at all timepoints, the change from baseline in PCOSQ was higher in EA than in SA. At 12-week treatment, the between-group difference was 10.51 (95% CI, 4.52 to 16,50); at 24-week follow-up, the between-group difference was 14.47 (95% CI, 7.48 to 21.47) (**Table 2**; **Supplementary Figure S2**).

### 3.4 Safety

The severity of adverse events was evaluated using the Common Terminology Criteria for Adverse Events (CTCAE) Version 5.0. Three patients in the EA group experienced skin bruising after acupuncture. Bruising and local discomfort were classified as Grade 1 (mild), defined as transient symptoms requiring no medical intervention. The bruising in all three patients resolved spontaneously within one week. No adverse events occurred in SA group during the entire treatment period. None of the subjects who experienced adverse events withdrew from the study, and the safety of the treatment was good in both groups.

### 3.5 Blinding

To evaluate the effectiveness of blinding implementation, participants were asked to guess their treatment group allocation at the end of the study (**Table 3**). In the EA group (n=53), 39.6% of participants correctly guessed their group allocation, 20.8% incorrectly guessed they were in the SA group, and 39.6% reported being unsure. In the SA group (n=53), 30.2% made correct guesses, 22.6% incorrectly guessed they were in the EA group, and 47.2% were unsure. The Blinding Index for the two groups was 0.313 (95% CI: -0.024 to 0.649) and 0.143 (95% CI: -0.226 to 0.511), respectively. The 95% confidence intervals of both groups included 0, indicating successful implementation of blinding (**Table 3**).

**Table 3:** Comparison of blinding assessment outcomes between the two groups.

Guess of treatment group, n (%)	EA (n=53)	SA (n=53)
EA group	21 (39.6)	12 (22.6)
SA group	11 (20.8)	16 (30.2)
Unsure	21 (39.6)	25 (47.2)
Blinding index (95%CI)	0.313 (-0.024, 0.649)	0.143 (-0.226, 0.511)

## 4 Discussion

In this randomized controlled trial investigating the effects of EA in patients with obese PCOS, EA treatment significantly reduced patients’ BMI and improved associated metabolic levels compared with SA. There were few adverse events related to EA were low. EA could be a valuable adjunctive therapy to promote recovery in patients with obese PCOS.

Acupuncture is a promising approach to metabolic regulation in patients with PCOS and helps to significantly reduce complications. According to TCM meridian theory and previous studies, Guanyuan (RN4), Uterus (EX-CA1), and Guilai (ST29) are the points most often chosen to regulate reproductive and metabolism functions. They both have significant efficacy in improving endocrine disruption and reproductive dysfunction in obese PCOS patients (20). In addition, studies have shown (21) that acupuncture at the ST29 may promote the secretion of gastric acid, pepsin, and other digestive juices, increase the activity of digestive enzymes in the gastrointestinal tract, and enhance the digestion and emptying of the gastrointestinal tract through the neural-humoral regulatory mechanism. In accordance with the principle of Sanyinjiao (SP6), the three meridians of the liver, spleen and kidney meet at this point. It exerts a regulatory effect on gastrointestinal transport and transformation and on hormone balance. A study showed that SP6 can promote gastrointestinal peristalsis and secretion of digestive juices and regulate intestinal flora (22). Taixi (KI3), as a source point of the kidney meridian, can improve obesity caused by kidney deficiency by regulating kidney function, excreting water and waste products, and alleviating edema and fat accumulation triggered by metabolic malfunction in PCOS patients (23). It has been reported that stimulation of KI3 can regulate the HPOA and the hypothalamic-pituitary-adrenal axis, balance the disordered hormone levels in PCOS patients, and indirectly regulate lipid metabolism. In addition, adding electrical stimulation to traditional acupuncture can promote the browning of white fat, improve lipid metabolism disorders by inhibiting cholesterol transport, influencing triglyceride metabolism, and inhibiting hepatic lipid synthesis, and reduce the symptoms of obesity in PCOS (24–26). Therefore, this trial aims to achieve optimal results by combining traditional acupuncture with modern electrophysiological techniques and selecting a streamlined combination of acupoints.

Obesity is a common clinical manifestation of metabolic abnormalities in PCOS patients, and improving body composition indexes is crucial for alleviating PCOS symptoms (27). Our study demonstrated that EA exhibited superiority over the SA group in enhancing BMI, WHR, and BFM. This implies that EA effectively reduces body weight and optimizes fat distribution in patients. Regarding SMM, although the change in the EA group was not statistically significant, there was no marked reduction, indicating that EA reduces fat without causing muscle mass loss. Jiang Shiyu (28) found that EA combined with laser could lower BMI in obese PCOS patients. Similarly, Chen Jiaxin (29) showed that EA with acupoint burrowing was beneficial for improving obesity indexes in such patients. These findings align with our study results. The issue of lipid metabolism and glucose metabolism parameters disorders in PCOS patients has drawn widespread global attention among clinical researchers, with studies indicating that approximately 48.3% of PCOS patients exhibit abnormal metabolism (30–32). Our study found that the EA group showed a significant reduction in lipid profile markers including TC, TG, and LDL-C, as well as glucose metabolism parameters including FINS, FPG, and HOMA-IR index levels post-treatment, demonstrating a significant positive effect of EA on regulating metabolism indexes in obese PCOS patients. In contrast, the SA group showed no meaningful improvement, which strongly supports the role of EA as an effective adjunctive intervention for improving dyslipidemia and insulin resistance. Notably, one study indicated that no significant difference between the EA group and the SA group in improving various metabolic indicators among PCOS patients. However, in-depth research revealed that the reason may lie in the use of shallow needling at non-meridian points as SA controls in the trial. This resulted in the SA group not being entirely ineffective but instead producing some degree of acupuncture effect (33). Another study indicated that four months after baseline visits, the acupuncture group demonstrated less efficacy than the metformin group in improving HOMA-IR. However, the metformin group experienced more frequent gastrointestinal side effects, including diarrhea, nausea, loss of appetite, fatigue, vomiting, and stomach discomfort(31.6%, 13.2%, 11.4%, 8.8%, 14.0% and 8.8%, respectively). The acupuncture group only developed bruising(14.9%) (34). In the present study, after 12 weeks of intervention, HOMA-IR in the EA group was significantly reduced compared with the baseline, and its efficacy was non-inferior to that of the SA control group. In contrast, the aforementioned previous study demonstrated that the acupuncture group was less effective than the metformin group in improving HOMA-IR. The core difference between the two studies lies in the efficacy positioning of the intervention measures: the present study focuses on the specific intervention effect of EA relative to the sham control, while the previous study emphasizes the direct efficacy comparison between acupuncture and a first-line drug. Differences in the core objectives of the study designs render the results of the two studies not directly comparable. Sex hormone levels are a main quantitative indicator of endocrine imbalance in obese PCOS patients. PCOS patients typically present with hyperandrogenemia, significantly elevated T levels compared to normal women, reduced E2 concentrations, increased PRL, and abnormally elevated LH/FSH ratios (35). Our study revealed a significant reduction in PRL and T levels and a significant increase in FSH and E2 levels in the EA group post - treatment. This indicates that EA can alleviate hyperandrogenism symptoms, promote estrogen level recovery, correct sex hormone imbalances in obese PCOS patients, and positively impact their reproductive function. The PCOSQ results show that EA has a significant positive effect on patients’ quality of life. It can enhance physical comfort, psychological well-being, and social functioning. This is highly significant for the comprehensive treatment and long-term management of obese PCOS patients.

PCOS patients often present with thin endometrium, enlarged OV, and irregular menstrual cycles. These symptoms are particularly common in obese PCOS patients and are closely linked to reduced fertility. In the field of PCOS, restoration of the menstrual cycle from oligomenorrhea/amenorrhea typically >35 days or absence of menstruation to the normal range 21–35 days constitutes a core clinical endpoint (36). During the late follicular phase or induced ovulation, an endometrial thickness <7 mm is generally defined as “thin endometrium,” which is associated with a decreased implantation rate. Therefore, increasing the endometrial thickness from below this threshold to ≥7–8 mm holds clear clinical significance, particularly for patients planning pregnancy (37). One of the ultrasonic diagnostic criteria for PCOS is a unilocular ovarian volume >10 cm³ excluding cysts and dominant follicles (38). A reduction in ovarian volume after treatment reflects improvements in the polycystic ovarian morphology and a potential decrease in the number of antral follicles. Although there is no single “curative” volume threshold, a reduction in ovarian volume to below 10 cm³ can be regarded as the normalization of ovarian morphology. Addressing these issues can significantly improve quality of life and long-term health outcomes in PCOS patients. Our study demonstrates that EA effectively promotes endometrial growth and development, helps the ovaries return to normal size and function, and shortens the menstrual cycle, making it more regular. These structural and functional ameliorations may enhance fertility potential in PCOS patients. Although EA has shown effectiveness in improving endometrial thickness, OV, and days of menstrual cycle, no significant difference was observed in comparison with the SA group. This may be related to possibly smaller sample sizes or greater individual differences.

Constrained by the existing data recording system, this study has not yet systematically quantified the consistency of participants’ intervention engagement. Consequently, it was unable to conduct an in-depth analysis of the potential moderating effect of adherence levels on intervention outcomes. Although the successful implementation of blinding has reduced certain biases, the lack of quantitative adherence data means that the potential impact of between-group differences in participation on the observed intervention effects cannot be fully ruled out. In future studies, while maintaining the blinding design, we will further incorporate multi-dimensional quantitative indicators such as objective interaction data from the WeChat platform, intervention implementation logs, and structured adherence questionnaires. This will enable more precise differentiation between the direct therapeutic effects of electroacupuncture and the indirect effects exerted through improved participation, thereby providing more robust evidence-based support for the in-depth validation of research conclusions and the optimization of clinical intervention protocols.

Although EA has demonstrated multiple clinical advantages for patients with obese PCOS, its underlying mechanism of action has not yet been fully elucidated. Future studies should integrate modern biological technologies, such as gene expression analysis and proteomics, to further explore the specific molecular mechanisms through which EA regulates endocrine function and metabolism.

## 5 Conclusions​

1. Both the EA group and SA group could reduce the BMI of patients with obese PCOS, but the efficacy of the EA group in reducing BMI was superior to that of the SA group at the 12th week of treatment and the 12th week of follow-up;s
2. The EA group exhibited better effects than the SA group in improving body composition (reducing WHR and BFM), regulating glucose metabolism (reducing FPG, FINS, and HOMA-IR), improving lipid metabolism (reducing TC, TG, and LDL-C), and regulating sex hormone levels (reducing PRL and increasing E2). This suggests that electroacupuncture has significant therapeutic advantages in improving the overall metabolic and endocrine status of patients with obese PCOS;n
3. Both the EA group and SA group could increase the thickness of the endometrium, reduce OV, and shorten the menstrual cycle of obese PCOS patients, but there was no statistically significant difference between the two groups.
4. At all treatment and follow-up timepoints, the change from baseline in PCOSQ was higher in EA than in SA. And this benefit became more significant with the passage of time.

## 6 Limitations

1. The current study design involves a three-month short-term intervention followed by a three-month follow-up, lacking long-term follow-up data. Future research should extend both the treatment and follow-up periods to evaluate the sustained effects of electroacupuncture therapy and its long-term impact on patients’ body composition, reproductive function, and metabolic status.
2. As a single-center, small-sample clinical study, this research has certain limitations. To enhance the reliability and generalizability of the findings, future studies should conduct multi-center, large-sample RCTs to verify the synergistic effects of EA combined with other therapies, and expand its clinical application scenarios. This will provide more effective and comprehensive treatment options for patients with PCOS.
3. The exact mechanism of action underlying electroacupuncture therapy remains incompletely understood. Future research should integrate modern biological techniques, such as gene expression analysis and proteomics, to thoroughly investigate the specific molecular mechanisms by which electroacupuncture modulates endocrine and metabolic functions.
4. The study population was limited to Chinese obese women with PCOS, which may restrict the generalization of the findings to other ethnic groups, non-obese PCOS patients, or those with comorbid type 2 diabetes mellitus. Future studies involving diverse populations are warranted to validate the effectiveness of the current results.

## Data Availability

The original contributions presented in the study are included in the article/**Supplementary Material**. Further inquiries can be directed to the corresponding authors.

## Supplementary material

Supplementary Data Sheet 1 Explanations on the details of the study protocol.​

Supplementary Figure 1 Diagram of acupuncture points;

Supplementary Figure 2 Statistical charts of all outcome indicators;

Supplementary Table 1 Acupoints Used in the Treatment;

Supplementary Table 2 Time nodes of the study design.
