# Supplementary material for: Effect of electroacupuncture on metabolic level and quality of life in patients with obese polycystic ovary syndrome: a randomized controlled trial
Source: Front Endocrinol (Lausanne). 2026 Jan 6;16:1723419. doi: 10.3389/fendo.2025.1723419 (PMC12815721; doi:10.3389/fendo.2025.1723419)
Supplement: Supplementary Data Sheet 1 — Explanations on the details of the study protocol.​ [file DataSheet1.docx]

Supplementary Material

# Supplementary Data Sheet 1

## Health Management Recommendations

# Diet​: A low-sugar, low-fat, and high-fiber diet is recommended; increase the intake of whole grains, vegetables, fruits, and high-quality protein; adopt a pattern of small and frequent meals; avoid high-sugar, high-fat foods and refined carbohydrates; limit the consumption of alcohol and coffee.​

# Exercise: Engage in at least 150 minutes of moderate-intensity aerobic exercise or 75 minutes of high-intensity exercise per week, such as brisk walking, jogging, swimming, etc.; perform strength training twice a week; it is recommended to exercise 30–60 minutes after meals.​

# Daily Behavioral Habits: Maintain a regular daily routine, ensure 7–8 hours of sleep every night, avoid bad habits such as staying up late, prolonged sitting, and smoking; keep a positive mindset, and monitor physical indicators regularly.

## **Basis for Electroacupuncture Parameters**

As a therapeutic method combining traditional acupuncture with modern electrophysiological technology, electroacupuncture therapy has demonstrated unique application value in clinical practice. Its core mechanism lies in applying electrical stimulation with specific frequency, waveform, and intensity to acupuncture needles inserted into specific acupoints via an electroacupuncture instrument. Studies have shown that electroacupuncture stimulation at specific acupoints can improve insulin resistance in polycystic ovary syndrome (PCOS) rats by promoting the browning of white adipose tissue. Additionally, electroacupuncture stimulation can regulate the body's metabolic function: it improves lipid metabolism disorders, enhances insulin sensitivity, and alleviates PCOS-related obesity through multiple pathways, such as inhibiting cholesterol synthesis, enhancing reverse cholesterol transport, affecting triglyceride metabolism, and suppressing hepatic lipid synthesis. It can also effectively regulate the human endocrine system, restore the normal function of the hypothalamic-pituitary-ovarian axis (HPOA), improve sex hormone imbalance, correct abnormally high androgen levels, regulate the menstrual cycle, and increase clinical ovulation and pregnancy rates. Owing to its comprehensive regulatory effects on multiple systems and targets of the body, electroacupuncture—this safe and effective novel therapeutic approach—has been widely applied in the clinical treatment of PCOS patients, opening up a new therapeutic pathway and bringing hope for recovery to PCOS patients.​

The Expert Consensus on the Application of Electroacupuncture and Transcutaneous Electrical Acupoint Stimulation in Reproductive Medicine clearly states that continuous-wave 2Hz low-frequency electroacupuncture is the mainstream choice in PCOS clinical research. 2Hz low-frequency electroacupuncture can promote the production of β-endorphin. As a neurotransmitter, β-endorphin can inhibit the release of gonadotropin-releasing hormone (GnRH). When GnRH release decreases, the blood levels of follicle-stimulating hormone (FSH) and luteinizing hormone (LH) secreted by the pituitary gland decrease accordingly. By regulating this endocrine axis, 2Hz low-frequency electroacupuncture helps restore ovulatory function and improve endocrine disorders, thereby exerting a certain improvement effect on obesity caused by endocrine imbalance. Furthermore, 2Hz low-frequency electroacupuncture contributes to reducing the activity of the c-Jun N-terminal kinase (JNK) pathway in skeletal muscle, inhibiting the serine phosphorylation of insulin receptor substrate 1 (IRS-1), promoting insulin signaling transduction, improving insulin resistance, and lowering blood glucose.​

Results from animal experiments have shown that low-frequency electroacupuncture can promote the browning of white adipose tissue and activate brown adipose tissue in PCOS rats, achieving weight loss effects. It also improves reproductive disorders in these rats, inducing ovulation after acupuncture, while simultaneously enhancing glucose tolerance, insulin tolerance, and indicators related to lipid metabolism and liver function. In a clinical study conducted by Gao Fei, PCOS patients with obesity received 2Hz low-frequency electroacupuncture treatment. After treatment, the patients' blood glucose, insulin levels, and fat metabolism-related indicators were measured. It was found that the patients' insulin resistance index decreased, and the oxidation rate of fatty acids in the blood increased. This indicates that low-frequency electroacupuncture can promote the catabolism of fat, contributing to weight reduction. In addition, low-frequency electroacupuncture may further inhibit fat accumulation by regulating the expression of relevant genes in adipose tissue, affecting the differentiation and proliferation of adipocytes, thereby achieving the effect of improving obesity.

## **Basis for Establishing the Sham Electroacupuncture Group**

This study aims to verify the therapeutic effects of electroacupuncture on body mass index (BMI), body composition, glucose-lipid metabolism, sex hormones, and menstrual cycle in obese patients with PCOS. Since acupuncture therapy may be interfered by non-specific factors such as psychological suggestion and patient expectations, which could affect the judgment of therapeutic effects, a sham electroacupuncture group was set up. This allows distinguishing these non-specific factors from the actual therapeutic effects of electroacupuncture, thereby accurately determining whether electroacupuncture exerts a substantial impact on the aforementioned symptoms and ensuring that the study results truly reflect the specific therapeutic effects of electroacupuncture.​

In this study, the Streitberger needle— the most commonly used placebo needle in clinical randomized controlled trials—was adopted for comparison, differing from traditional acupuncture needles. This nested, slidable flat-tip Streitberger needle creates the visual illusion of the needle body penetrating the skin and the tactile sensation of acupuncture, which is similar to the experience of receiving real acupuncture. This helps maintain the consistency and comparability of the study, avoiding differences in the psychological state of subjects caused by varying treatment experiences (which might otherwise affect the study results). However, the needle tip does not actually penetrate the skin, and thus, no effective acupuncture effect is produced.​

In addition, after connecting the electrodes to the Streitberger needle, although the indicator light of the electroacupuncture instrument remains on, the current intensity is not activated. This largely ensures that subjects cannot distinguish the grouping of each group from the appearance, thereby creating conditions for the smooth implementation of blinding. It effectively reduces subjective bias caused by subjects knowing their group assignment, thus guaranteeing the objectivity and scientificity of the study results.​

Furthermore, the establishment of the sham electroacupuncture group in this study strictly adheres to the principles of medical ethics and fully reflects the protection of subjects' rights and interests. The sham electroacupuncture group simulates part of the experience of real electroacupuncture therapy without causing any invasive harm. The special needles used in the sham electroacupuncture group cannot penetrate the skin, fundamentally eliminating all potential physical injury risks that may be caused by acupuncture. Moreover, only the electroacupuncture instrument is connected without activating the current intensity, so there are no potential adverse effects caused by electrical stimulation. Therefore, the establishment of the sham electroacupuncture group not only does not reduce safety but also further demonstrates the rigor of the entire study in ensuring the safety of subjects through comparison, strongly upholding the core principle of "non-maleficence" in medical ethics.

**1.4 Acupoint Selection Rationale Based on Traditional Chinese Medicine Theory**

Guanyuan (RN4) serves as a crossing point of the Conception Vessel (RN) and the three Yin meridians of the foot (Spleen, Liver, Kidney). It functions to tonify primordial qi, warm yang, and consolidate the root.

Uterus (EX-CA1) is classified as an extraordinary point, commonly utilized for managing gynecological conditions including irregular menstruation, dysmenorrhea, and endometriosis.

Guilai (ST29) belongs to the Stomach Meridian of Foot-Yangming. The Stomach and Spleen Meridians are internally-externally related; thus, this acupoint can disperse stagnated qi in the spleen and stomach, promote the digestion and transformation of food nutrients, and reduce phlegm-dampness accumulation.

Sanyinjiao (SP6) is a crossing point of the Spleen, Liver, and Kidney Meridians. It strengthens the spleen and replenishes qi to resolve dampness, nourishes blood and invigorates blood circulation to unblock meridians, and regulates the liver and kidney. It exerts a favorable regulatory effect on endocrine disorders, irregular menstruation, and infertility.

Taixi (KI3), as the source point of the Kidney Meridian of Foot-Shaoyin, nourishes kidney yin, warms the kidney, and regulates menstruation.

Adjunct Acupoints for Syndrome Differentiation

Kidney Deficiency with Blood Stasis Type

Qihai (RN6) is a gathering site of innate primordial qi. It warms yang to dispel cold, regulates qi movement, and replenishes primordial qi, demonstrating significant efficacy in alleviating symptoms such as qi deficiency fatigue, irregular menstruation, and dysmenorrhea.

Xuehai (SP10) possesses dual effects of invigorating blood circulation to remove stasis and nourishing blood. Acupuncture at this acupoint improves blood circulation, promotes fluid metabolism, unblocks qi and blood flow, restores regular menstruation, thereby regulating endocrine function and alleviating obesity caused by qi-blood stagnation.

Kidney Deficiency with Liver Stagnation Type

Taichong (LR3), the source point of the Liver Meridian of Foot-Jueyin, soothes liver stagnation, regulates qi, and invigorates blood circulation. It alleviates symptoms such as chest distress, hypochondriac pain, and irregular menstruation induced by kidney deficiency combined with liver stagnation.

Qimen (LR14), the front-Mu point of the Liver Meridian, directly regulates liver qi and blood, and disperses stagnated liver qi. Smooth liver qi facilitates emotional regulation and improves gynecological disorders triggered by emotional distress. Liver stagnation often impairs the digestive function of the spleen and stomach; stimulation of Qimen (LR14) soothes the liver and strengthens the spleen, restores normal digestive function, promotes the absorption and distribution of nutrients, prevents fluid retention and phlegm-dampness accumulation, and reduces obesity.

Spleen Deficiency with Phlegm-Dampness Type

Yinlingquan (SP9), the he-sea point of the Spleen Meridian of Foot-Taiyin, excels in treating "dampness syndromes". It strengthens the spleen to promote diuresis and resolves phlegm-dampness by regulating spleen qi, thereby unblocking the triple burner and facilitating the normal distribution and excretion of body fluids. Unobstructed triple burner function ensures smooth fluid metabolism, preventing obesity caused by fluid retention, promoting overall metabolism, and alleviating symptoms such as lower limb edema. It fundamentally eliminates the root cause of phlegm-dampness accumulation, effectively improving obesity, limb heaviness, and loose stools resulting from spleen deficiency with excessive dampness.

Fenglong (ST40) belongs to the Stomach Meridian of Foot-Yangming and is a key acupoint for resolving phlegm. Electroacupuncture stimulation at Fenglong (ST40) enhances the digestive and metabolic functions of the spleen and stomach, strengthens the metabolism and distribution of fluids, promotes the transformation and excretion of phlegm-dampness, reduces its accumulation in the body, and thereby achieves weight loss.

Detailed information on the meridian attribution and localization of the acupoints is provided in Supplementary Table 1: Acupoints Used in the Treatment.

**1.5 Results of Body Weight-Related Outcome Measures**

Comparison of body weight-related outcome measures between the two groups after 12 weeks of treatment revealed that the EA group exhibited significantly superior performance to the SA group in terms of absolute body weight reduction, percentage of body weight loss, and the rate of achieving ≥5% body weight loss. All intergroup differences were statistically significant (all P<0.001), with detailed data presented in Supplementary Table 3.

Body Weight Change: After 12 weeks of treatment, the EA group (n=53) achieved a mean body weight reduction of (6.40±2.43) kg, while the SA group (n=53) had a mean reduction of (2.47±1.92) kg. Intergroup comparison showed that the absolute body weight reduction in the EA group was significantly greater than that in the SA group, with a mean difference of 3.93 kg (95% confidence interval [CI]: 3.09 to 4.78 kg), and this difference was statistically significant (t=9.27, P<0.001).

Percentage of Body Weight Loss: The mean percentage of body weight loss in the EA group after 12 weeks was (8.56±2.61)%, which was significantly higher than the (3.33±2.28)% observed in the SA group. The intergroup difference was 5.23 percentage points (95% CI: 4.28 to 6.17 percentage points), and this difference was statistically significant (t=10.98, P<0.001), indicating a marked relative advantage of EA in promoting weight loss.

Rate of Achieving ≥5% Body Weight Loss: From the perspective of clinically effective population coverage, 51 patients in the EA group (96.2% of the group) achieved the standard of ≥5% body weight loss, whereas only 7 patients in the SA group (13.2% of the group) met this criterion. Intergroup comparison demonstrated that the achievement rate in the EA group was significantly higher than that in the SA group, with a statistically significant difference (χ²=73.71, P<0.001).

In this study, independent samples t-test was used for intergroup comparison of continuous variables, including body weight change and percentage of body weight loss, while the chi-square test was applied for categorical variables such as the rate of achieving ≥5% body weight loss. The significance level was set at 0.05 for all analyses. Notably, the 95% CIs of all outcome measures did not include 0, which further verified the authenticity and reliability of the observed intergroup differences. Collectively, these results indicate that EA therapy has a definite advantage in improving body weight-related indicators in patients with obese PCOS.

# Supplementary Figures and Tables

##
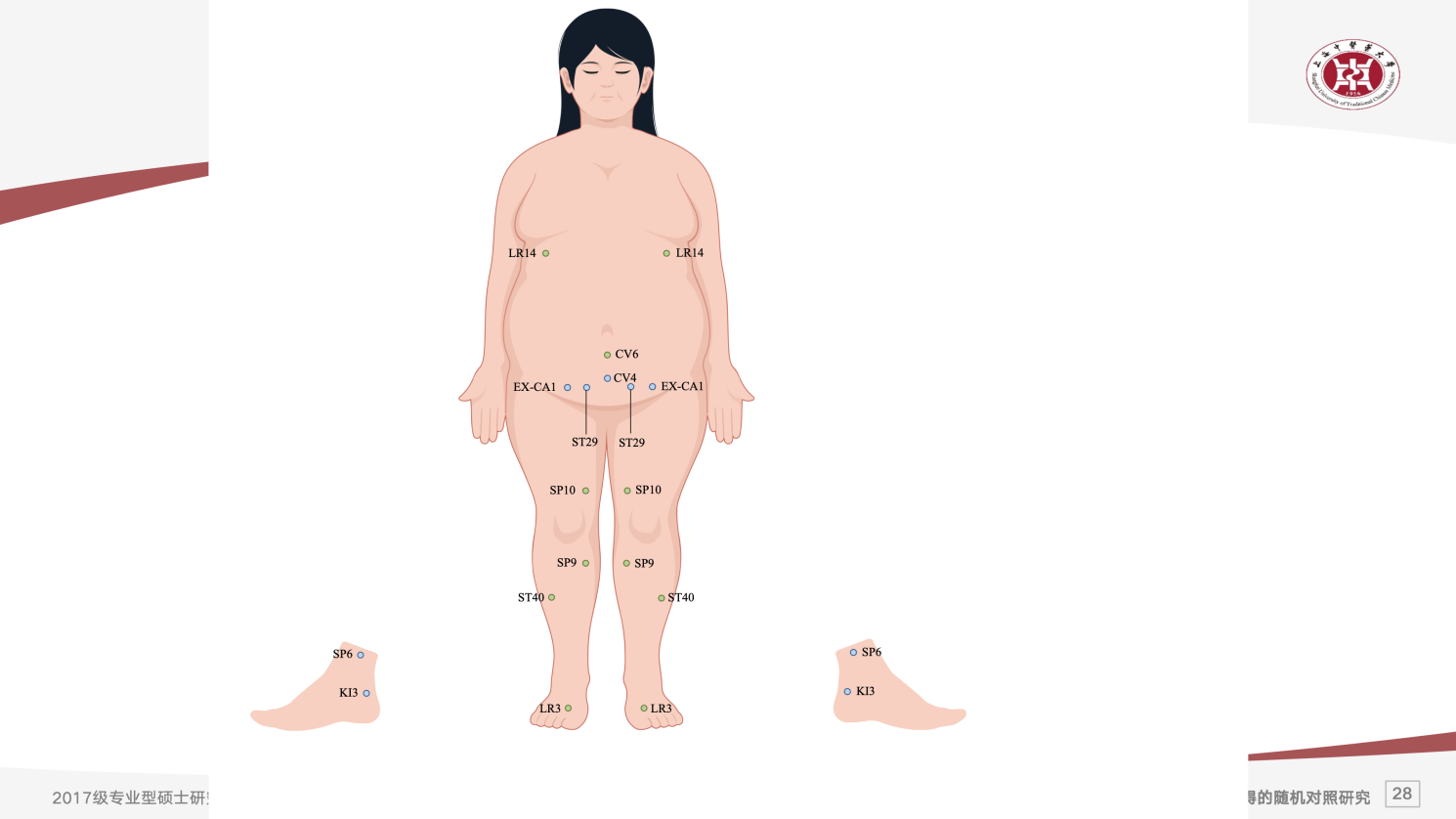


## **Supplementary Figure 1. Diagram of acupuncture points.** Blue indicates the main acupoints, and green indicates the compatible acupoints.

**
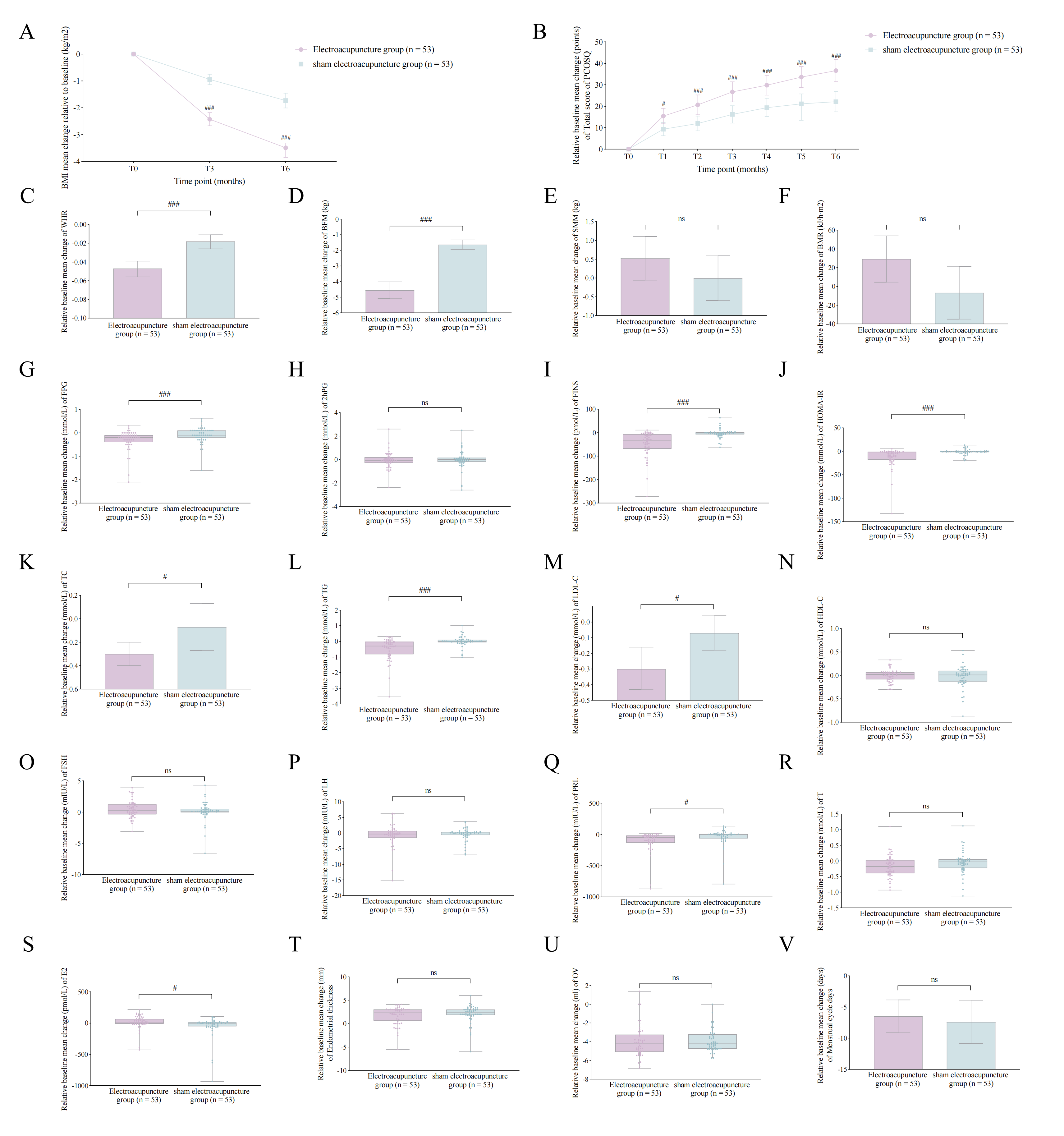
**

**Supplementary Figure 2. Statistical charts of all outcome indicators.**T0 denotes pre-treatment, T3 denotes the 3rd month of treatment, and T6 denotes the 3rd month of follow-up. For inter-group comparisons, #*P*<0.05, ##*P*<0.01, and ###*P*<0.001.

**Supplementary Table 1. Acupoints Used in the Treatment**

| Acupoints | Meridian Attribution of Acupoints | Location | |
| --- | --- | --- | --- |
| Main Acupoints | | |  |
| Guanyuan (RN4) | Conception Vessel | In the lower abdomen, on the anterior midline, 3 inches below the umbilical cord. | |
| Uterus (EX-CA1) | Extraordinary Point | In the lower abdomen, 3 inches off the anterior midline, 4 inches below the umbilicus. | |
| Guilai (ST29) | Stomach Meridian of Foot-Yangming | In the lower abdomen, 2 inches beside the anterior median line, 4 inches below the umbilicus. | |
| San Yinjiao (SP6) | Spleen Meridian of Foot-Taiyin | On the medial side of the shank, 3 cun directly above the tip of the medial malleolus, on the posterior border of the medial aspect of the tibia. | |
| Taixi (KI3) | Kidney Meridian of Foot-Shaoyin | Back of the inner ankle, in the depression between the tip of the inner ankle and the Achilles tendon. | |
| Match Acupoints | | | |
| Kidney Deficiency and Blood Stasis Type | | | |
| Qihai(RN6) | Conception Vessel | In the lower abdomen, on the anterior midline, 1.5 inches below the umbilical cord. | |
| Xuehai(SP10) | Spleen Meridian of Foot-Taiyin | Medial thigh, 2 inches above the medial aspect of the base of the patella, at the elevation of the medial head of the quadriceps muscle. | |
| Kidney Deficiency and Liver Depression | | | |
| Taichong(LR3) | Liver Meridian of Foot-Jueyin | On the dorsum of the foot, in the depression before the union of the 1st and 2nd phalanges. | |
| Qimen(LR14) | Liver Meridian of Foot-Jueyin | On the chest, 4 inches off the anterior midline, at the 6th intercostal space. | |
| Spleen Deficiency Phlegm Dampness | | | |
| Yin Lingquan(SP9) | Spleen Meridian of Foot-Taiyin | On the medial side of the shank, on the lower border of the medial condyle of the tibia. | |
| Fenglong(ST40) | Stomach Meridian of Foot-Yangming | On the anterolateral side of the lower leg, 8 inches above the tip of the outer ankle, 2 transverse fingers (middle finger) from the anterior edge of the tibia. | |

**Supplementary Table 2. Time nodes of the study design**

| Timepoint | Enrollment Week -1 | Allocation Week 0 | Treatment | | | Follow-up | | |
| --- | --- | --- | --- | --- | --- | --- | --- | --- |
|  |  |  | Week 4 | Week 8 | Week 12 | Week 16 | Week 20 | Week 24 |
| Enrollment | × |  |  |  |  |  |  |  |
| Informed consent | × |  |  |  |  |  |  |  |
| Baseline assessments |  | × |  |  |  |  |  |  |
| Health education | × |  |  |  |  |  |  |  |
| Randomization |  | × |  |  |  |  |  |  |
| Intervention |  |  | × | × | × |  |  |  |
| Primary outcomes |  |  |  |  |  |  |  |  |
| BMI |  | × |  |  | × |  |  | × |
| Secondary outcomes |  |  |  |  |  |  |  |  |
| Body composition analysis |  | × |  |  | × |  |  |  |
| Glycolipid metabolism indexes |  | × |  |  | × |  |  |  |
| Sex hormone indexes |  | × |  |  | × |  |  |  |
| Endometrium and ovulation |  | × |  |  | × |  |  |  |
| Menstrual cycle |  |  | × | × | × | × | × | × |
| PCOSQ |  | × | × | × | × | × | × | × |
| Adverse event monitoring |  |  | × | × | × |  |  |  |

**Supplementary Table 3. Comparison of Observation Indicators Related to Body Weight After Treatment Between Two Groups**

| Observation Indicators | EA (n=53) | SA (n=53) | Difference Between Groups  *t/*$x^{2}$ | (95%CI) | *P* |
| --- | --- | --- | --- | --- | --- |
| Body Weight Change(kg,x$\pm$s) | | | | | |
| Week 12 | 6.40$\pm$2.43 | 2.47$\pm$1.92 | 9.27 | (3.09,4.78) | <.001 |
| Percentage of Body Weight Loss(%,x$\pm$s) | | | | | |
| Week 12 | 8.56$\pm$2.61 | 3.33$\pm$2.28 | 10.98 | (4.28,6.17) | <.001 |
| Proportion of Participants Achieving ≥5% Body Weight Loss(n,%) | | | | | |
| Week 12 | 51(96.2%) | 7(13.2%) | 73.71 |  | <.001 |
